# Research on the screening mechanisms of composite vibrating screens based on discrete elements

**DOI:** 10.1371/journal.pone.0293205

**Published:** 2023-10-19

**Authors:** Huarui Yang, Xuedong Ma

**Affiliations:** School of Mechanical Engineering and Automation, University of Science and Technology Liaoning, Anshan, Liaoning, China; Sivas Cumhuriyet University, TURKEY

## Abstract

To strengthen the screening efficiency of traditional vibrating screens, a new type of vibrating screen, namely the composite vibrating screen, has been proposed based on the Lissajous vibration synthesis theory. The working principles of composite vibrating screens have been explained. Numerical simulations of the sieving processes for such composite vibrating screens were carried out using the discrete element method. Compared with traditional linear vibrating screens, the force, stratification mechanisms, and throwing principles of the material on the screen’s surface were studied, and the vibrating screens’ material transportation and screening efficiency were analyzed. The results showed that with the existence of *xyz* three directions sub-vibrations of the composite vibrating screens, the material particle group is more diversified by the forces, the particle system is loose, the stratification effects are adequate, and the material is evenly distributed on the screen surfaces. Under the same vibration parameters, the composite vibrating screens’ screening efficiencies and material transportation capacities were better than those of linear vibrating screens. This work provides a necessary reference for the development and application of new composite vibrating screens.

## 1. Introduction

As a kind of granular material sorting equipment, vibrating screens are widely used in the mining, metallurgy, coal, medicine industries as well as other industries because of their simple structures and reliable sorting capabilities. According to the trajectory of the screen body’s movement, vibrating screens are mainly divided into the categories of linear, circular, and elliptical vibrating screens [1–3]. The screening processes of vibrating screens are mainly composed of four parts: feeding, stratification, sieving and discharge, among which the particle stratification effects directly determine the levels of the through sieve rate [4–8]. Stratification refers to the "Brazil nut" configuration of the particles on the screen surfaces, with larger particles at the tops and smaller particles at the bottoms. [9, 10]. The closer the small particles are to the screen’s surface, the easier it is for them to penetrate the sieves.

With the rapid development and maturation of numerical simulation technologies over recent years, scholars have been provided with faster, more economical, and more reliable research means. The discrete element method (DEM) is a computational numerical simulation method used to analyze particle motion behaviors and mechanical properties. It has been proven to be an effective numerical simulation technique for calculating the motion behaviors of discrete particles based on Newton’s second law. It has been widely applied in the field of discrete particulate matter spheres [11–14], such as in mining [15–17], agricultural [18, 19], and chemical engineering [20]. Many scholars have researched vibrating screen equipment using such numerical simulation techniques. For example, WU et al. [21] analyzed the effects of vibration parameters on the screening efficiency using a discrete unit method simulation and digitally fitted their results; YIN et al. [22] studied the relationship between the throwing index and screening efficiency, summarized the empirical formulas for both, and studied the screening efficiencies of elliptical vibrating screens, finding that elliptical vibrations had a strong treating ability; ZHAO et al. [23] established an orthogonal experiment to explore the effects of the vibration parameters on the screening effects and optimized the vibration parameters. However, most research has focused on discussing the vibration parameters and the optimization of the structures of linear, elliptical, and circular vibrating screens, so there has been a marked lack of breakthroughs related to the vibration modes of vibrating screens.

In 1857, Jules Antoine Lissajous proposed a theory concerning the synthesis of two mutually perpendicular simple harmonic vibrations. Both vertical circular vibrations and horizontal circular vibrations, when considered in polar coordinates, can be considered to be single modes of vibration that are defined by a pendulum angle and swing frequency. Considering this alongside Lissajous vibration synthesis theory, they can be considered composites of two mutually perpendicular one-dimensional simple harmonic motions, with the same frequencies and amplitudes and a phase difference of 90°. Elliptical vibrations are thus composites of two mutually-perpendicular one-dimensional simple harmonic vibrations of the same frequencies and different amplitudes. If the frequency ratios and phase differences of two one-dimensional simple harmonic vibrations are changed, more complex vibration curves can be synthesized.

This paper breaks down the vibration modes of traditional vibrating screens, and based on Lissajous vibration synthesis theory, synthesizes two one-dimensional simple harmonic vibrations with a mutual perpendicularity and frequency ratio of 1:2 into composite vibrations. MATLAB software was used to establish the mathematical model of the Lissajous vibration curve, while EDEM software was used to explore the mechanisms of composite vibrating screens in terms of the material throwing, material loosening and stratification, as well as the advantages it has in terms of the screening efficiency and transportation capacity. To lay the theoretical foundation for the future design of the composite vibrating screen.

## 2 Model setup

### 2.1 Mathematical model

According to Lissajous vibration synthesis theory, two one-dimensional simple harmonic vibrations that are mutually perpendicular and have a frequency ratio of 1:2 can be synthesized into a composite vibration in a plane determined by the two one-dimensional vibrations

$$\left\{\begin{array}{l} S_{1}=A_{1}\sin(\omega_{1}t+\varphi_{1})\\ S_{2}=A_{2}\sin(\omega_{2}t+\varphi_{2}) \end{array}\right.$$ (1)

Where *S*_1_, *S*_2_ are one-dimensional simple harmonic vibrations; *A*_1_, *A*_2_ are the amplitudes in mm, and *ω*_1_, *ω*_2_ are the angular velocities of the vibration in rad/s; *φ* is the phase in rad, and *φ*_1_-*φ*_2_ = 0.

**Fig 1:**
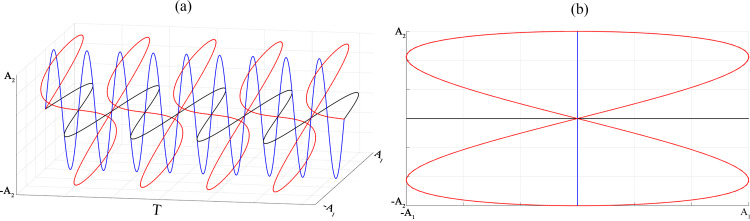
Composite vibration synthesis diagram and composite vibration trajectory diagram.

Two one-dimensional simple harmonic vibrations are synthesized into composite vibrations using the MATLAB software, and the composite vibration curve is shown in Fig 1(A); the blue curve and black curve are two mutually perpendicular simple harmonic vibration curves while the red curve is the composite vibration curve, and Fig 1(B) shows the composite vibration trajectory.

### 2.2 Physical model of particle motion

The particles are subjected to multiple effects of gravity, the tangential force distance, the normal force distance and friction during their motion. From this, the equation of motion for the *i* particle can be deduced as [22]:

$${\text{m}}_{i}\frac{dV_{i}}{dt}=F_{\text{g}}+F_{f}+\sum_{\text{j}=1}^{ni}\left(F_{n,ij}+F_{t,ij}\right)$$ (2)

$$I_{i}\frac{d\omega_{i}}{dt}=\sum_{j=1}^{ni}\left(T_{t,ij}+T_{r,ij}\right)$$ (3)

Where *m*_*i*_ is the mass of the particle in units of kg, *I*_*i*_ is the rotational inertia of the particle in units of kg•m^2^, *V*_*i*_ is the particle velocity in units of m/s, *ω*_*i*_ is the angular velocity of the particle in units of rad/s, *F*_*g*_ is the gravitational force in units of N, *F*_*f*_ is the frictional force in units of N, *F*_*n*,*ij*_ is the normal force in units of N, *F*_*t*,*ij*_ is the tangential force in units of N, *T*_*t*,*ij*_ is the tangential moment in units of N·m, and *T*_*r*_,_*ij*_ is the normal moment in units of N·m.

According to the Hertz contact theory, the normal force *F*_*n*,*ij*_ between particle *i* and particle *j* can be expressed as [23]:

$$F_{n,ij}=-\frac{4}{3}E*\sqrt{R*}\lambda_{n}{}^{\frac{3}{2}}n_{c}-\sqrt{\frac{5}{6}k_{n}m*}\frac{2\text{ln}\varepsilon }{\sqrt{{\text{ln}}^{2}\varepsilon +\pi^{2}}}(v_{n,ij}\cdot n_{c})n_{c}$$ (4)

Where E*, R* and m* are the equivalent elastic modulus, radius, and mass, respectively; *λ*_*n*_ is the normal overlap in units of m; *n*_*c*_ is the unit vector; *k*_*n*_ is the normal stiffness; *ε* is the elastic recovery coefficient; and *v*_*n*,*ij*_ is the relative velocity normal to particle *i* and particle *j* in units of m/s.

The tangential force *F*_*t*,*ij*_ can be deduced from Mindlin-Deresiewicz theory as [24]:

$$F_{t,ij}=-8G*\sqrt{R*\lambda_{\text{n}}\lambda_{t}}-\sqrt{\frac{5}{6}k_{t}m*}\frac{2\text{ln}\varepsilon }{\sqrt{{\text{ln}}^{2}+\pi^{2}}}(v_{t}{}_{,ij}\cdot n_{c})n_{c}$$ (5)

Where: G* is the equivalent shear modulus, *λ*_*t*_ is the tangential overlap in m, *k*_*t*_ is the tangential stiffness, and *v*_*t*,ij_ is the tangential relative velocity of particle *i* and particle *j* in units of m/s.

The tangential and normal moments are [25]

$$T_{t,ij}=n_{i}\cdot F_{t,ij}$$ (6)

$$T_{r,ij}=-\mu F_{n}{}_{,ij}n_{i}\omega_{i}$$ (7)

Where: *μ* is the rolling friction coefficient, *n*_*i*_ is the unit direction vector the center of mass of the particle *i* to the contact point, and *ω*_*i*_ the angular velocity unit vector at the contact point of particle *i*.

### 2.3 Vibrating screen motion analysis

**Fig 2:**
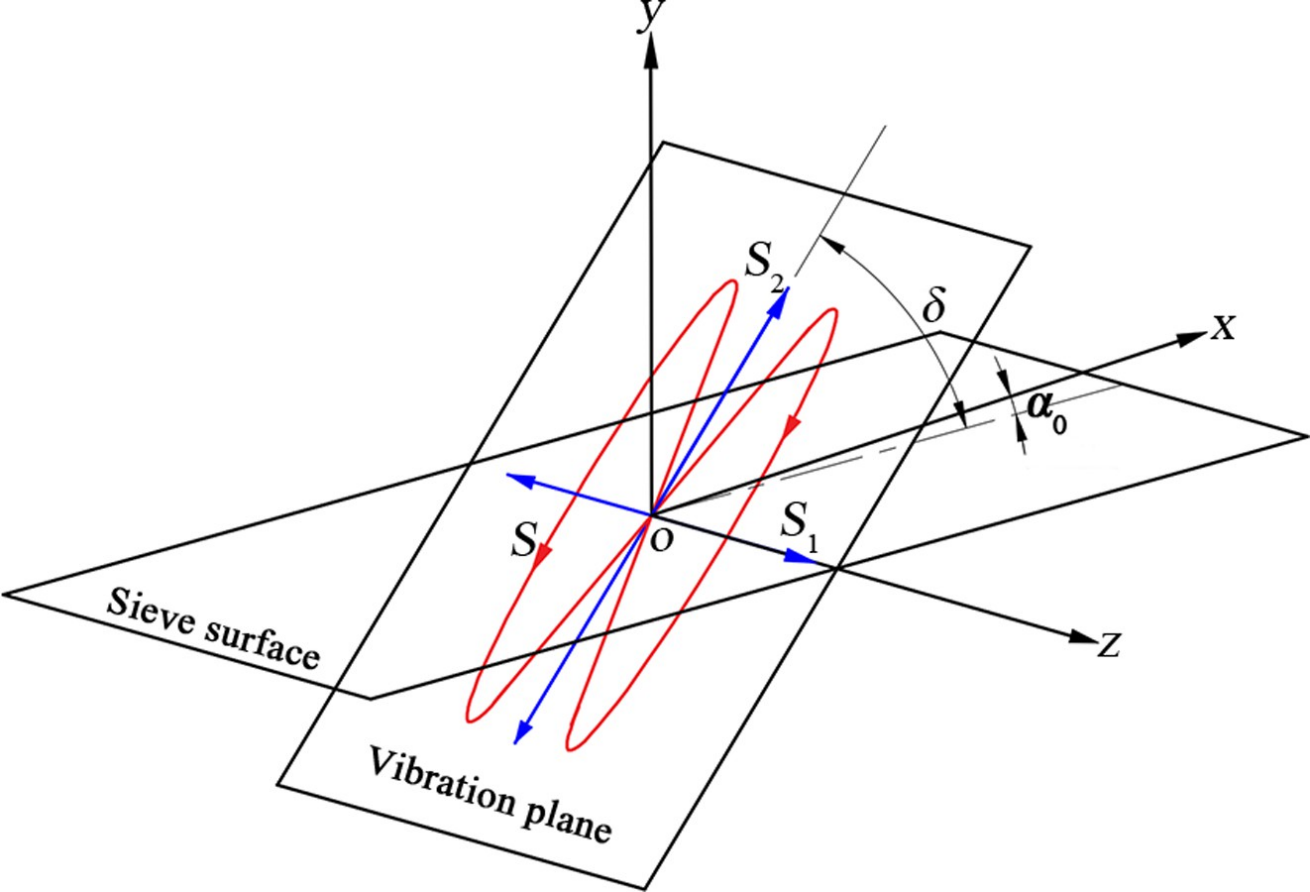
Schematic diagram of the movement of the screen’s surface.

According to the mathematical model, two simple harmonic vibrations perpendicular to each other with a frequency ratio of 1:2 are added to the barycenter of the vibrating body, so as to synthesize them into a composite vibration. As shown in Fig 2, *S*_1_ exhibits a simple harmonic vibration along the *z*-axis, while *S*_2_ exhibits a simple harmonic vibration along the direction of the angle *δ* of 45° with the screen’s surface, so a Lissajous composite vibration is synthesized in the plane at an angle of 45° with the screen’s surface, with the angle *δ* defined as the vibration direction angle. The inclination angle of the screen’s surface *α*_0_ is set as 3°, and the screen’s body moves along the Lissajous composite vibration curve *S*.

**Fig 3:**
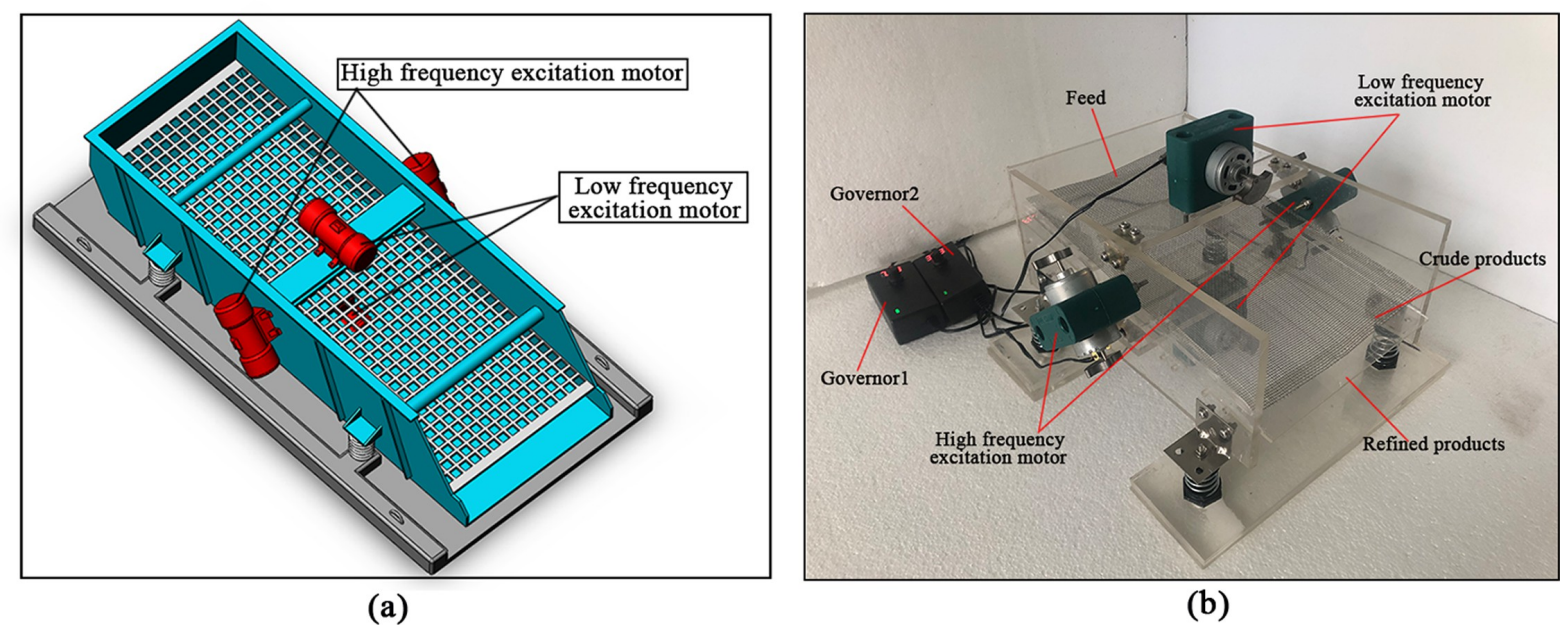
Composite vibrating screen model.

The composite vibrating screen can be realized using two pairs of excitation motors with different frequencies; the rotational speed ratio of the high-frequency excitation motor and low-frequency excitation motor is 2:1, and the two pairs of excitation motors are distributed symmetrically around the center of mass. Two low-frequency excitation motors are arranged symmetrically on the upper and lower sides of the screen box for equal speed reverse rotation, synthesizing *S*_1_. Two high-frequency excitation motors are arranged symmetrically on the left and right sides, and the excitation motors make an angle of 45° to the *x*-direction for equal speed reverse rotation to synthesize *S*_2_. The specific implementation is shown in Fig 3(A) and 3(B) provides a model of the composite vibrating screen, where said high-frequency excitation motor and low-frequency excitation motor are controlled by two speed governors. The excitation motor is distributed around the center of mass of the screen body, which effectively eliminates the torque generated by the excitation motor on the center of mass and avoids the torsional movement of the screen box.

**Fig 4:**
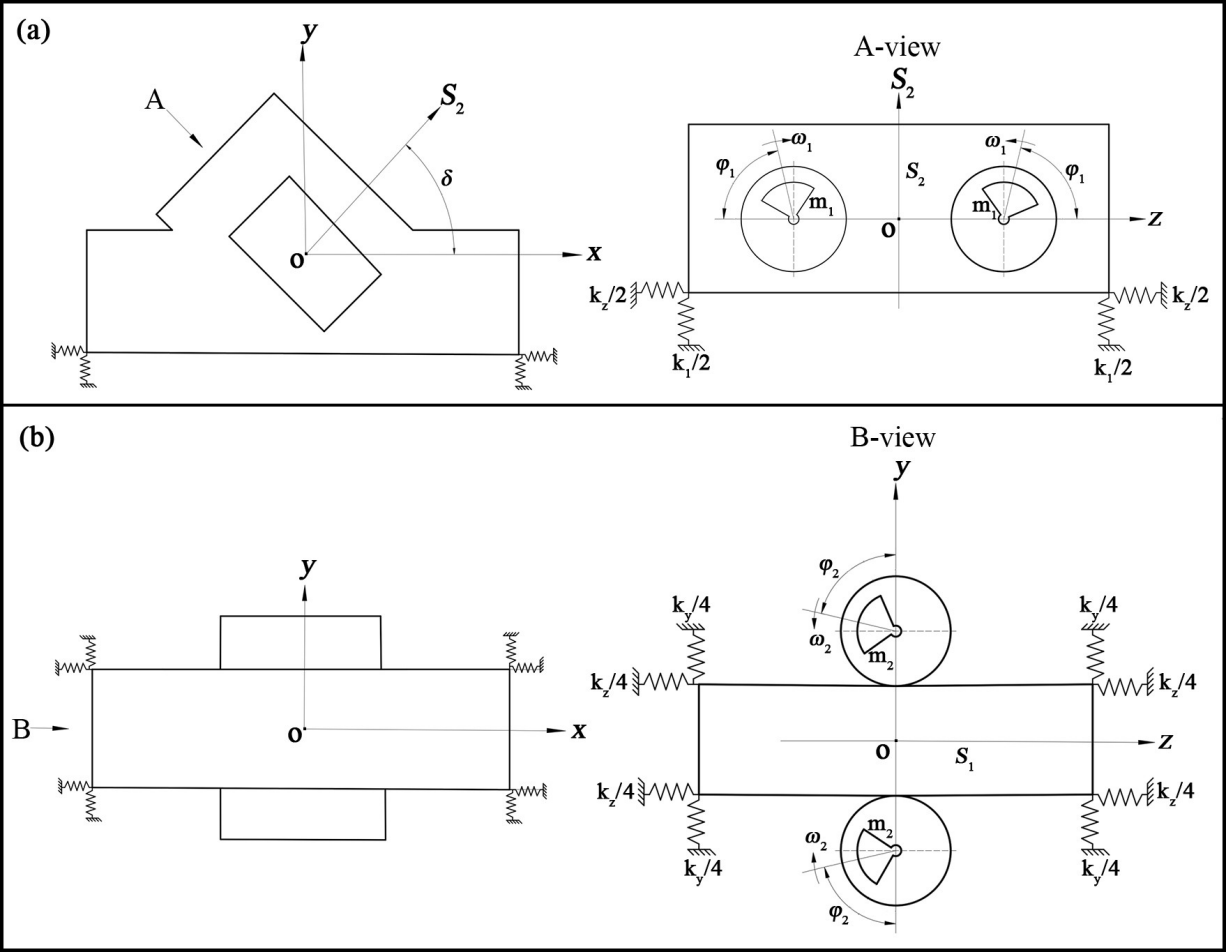
Simplified model of a composite vibrating screen.

The simplification of the screen box and the excitation motor leads to its schematic diagram, as shown in Fig 4. The two high-frequency excitation motor eccentric rotor masses are m_1_, and due to the two reverse equal-speed rotations, the two rotors generated by the excitation force in the *z*-direction cancel each other out in the *S*_2_ direction of their superposition, so it can be formed along the direction of *S*_2_ in the simple harmonic straight-line vibration, as shown in Fig 4(A); the two low-frequency excitation motor eccentric rotor mass are m_2_, both with the same reverse equal speed rotations, and the two rotors generated by the excitation force in the *y*-direction cancel each other out in their *z*-direction superposition, so it can be formed along the *z*-direction of the simple harmonic linear vibration *S*_1_, as shown in Fig 4(B).

By decomposing the excitation force in the *S*_2_ direction into both the *x* and *y* directions, two excitation forces can be obtained, from which a differential equation for the motion of the screen box can be obtained:

$$\left\{\begin{array}{l} M\ddot{x}+c_{x}\dot{x}+k_{x}x=m_{1}r_{1}\omega_{1}^{2}\text{sin}(\omega_{1}t+\varphi_{1})\text{cos}\delta \\ M\ddot{y}+c\dot{y}+k_{y}y=m_{1}r_{1}\omega_{1}^{2}\text{sin}(\omega_{1}t+\varphi_{1})\text{sin}\delta \\ M\ddot{z}+c_{z}\dot{z}+k_{z}z=m_{2}r_{2}\omega_{2}^{2}\text{sin}(\omega_{2}t+\varphi_{2}) \end{array}\right.$$ (8)

Where: M, m_1_, m_2_ are respectively the masses of the screen box, eccentric block 1, and eccentric block 2; $\ddot{x}$、$\dot{x}$、*x* are the acceleration, velocity and displacement of the screen box in the *x* direction; $\ddot{y}$, $\dot{y}$, *y* are the acceleration, velocity and displacement of the screen box in the *y* direction; $\ddot{Z}$, $\dot{Z}$, , *z* are the acceleration, velocity and displacement of the screen box in the *z* direction; *c* is the damping coefficient, *k*_*1*_ and *k*_*2*_ are the stiffness coefficients of the springs in the *S*_1_ and *S*_2_ directions, *r* is the eccentricity of the oscillator; and *ω*_*1*_ and *ω*_*2*_ are the phase angles of eccentric blocks 1 and 2.

The steady-state solution of the equations of motion can be found as:

$$\left\{\begin{array}{l} x(t)=A_{x}\sin(\omega_{1}t+\varphi_{x})\\ y(t)=A_{y}\sin(\omega_{1}t+\varphi_{y})\\ z(t)=A_{z}\sin(\omega_{2}t+\varphi_{z}) \end{array}\right.$$ (9)

Among them:

$$\begin{array}{l} \varphi_{x}=-\arctan\frac{c_{x}\omega_{1}}{k_{x}-M\omega_{1}^{2}}-\varphi_{1}\\ A_{x}=\frac{m_{1}r_{1}\omega_{1}^{2}}{k_{x}-M\omega_{1}^{2}}\cos\delta \sin\varphi_{x}\\ \varphi_{y}=-\arctan\frac{c_{y}\omega_{1}}{k_{y}-M\omega_{1}^{2}}-\varphi_{1}\\ A_{y}=\frac{m_{1}r_{1}\omega_{1}^{2}}{k_{y}-M\omega_{1}^{2}}\sin\delta \sin\varphi_{y}\\ \varphi_{z}=-\arctan\frac{c_{z}\omega_{2}}{k_{z}-M\omega_{2}^{2}}-\varphi_{2}\\ A_{z}=\frac{m_{2}r_{2}\omega_{2}^{2}}{k_{z}-M\omega_{2}^{2}}\cos\varphi_{z} \end{array}$$ (10)

From the steady state solution, it can be seen the sieve body is displaced in all three directions, *xyz*.

**Fig 5:**
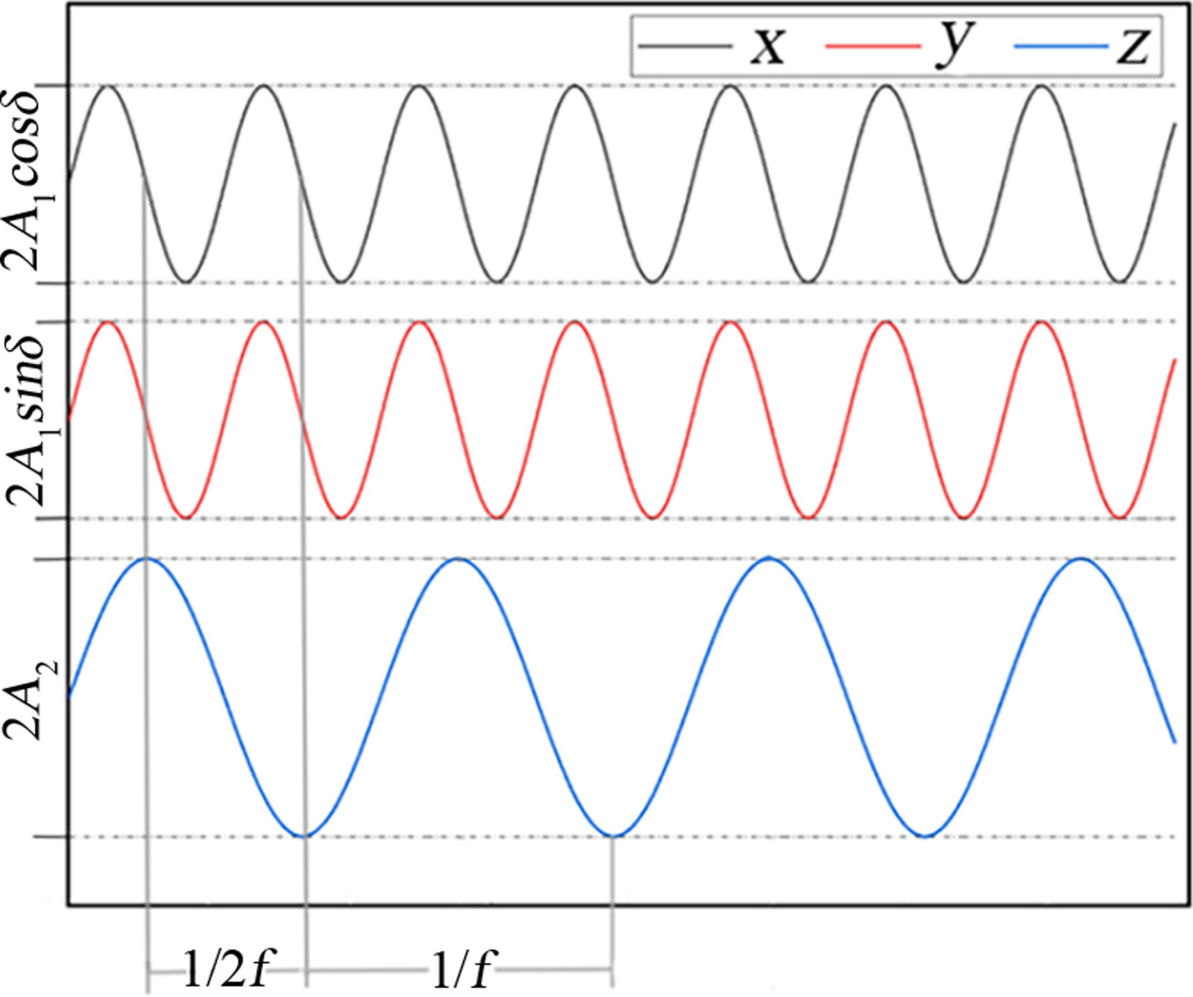
Projections of composite vibration curves in three directions *xyz*.

Projecting the composite vibration curve shown in Fig 1 in the direction of the *xyz* three coordinate axes, we can obtain the sub-vibrations along the three directions, as shown in Fig 5. It can be seen from Fig 5 that the projection of the composite vibration curve is three simple harmonic vibrations, which indicates that the sieve body in the *xyz* direction there are displacements, so the screen mesh can give the material to provide three directions of the excitation force. This also corresponds to Eq 9.

After the particles enter the sieve box, relying on friction and the impact force provided by the screen mesh to obtain their energy, and they generally undergo three processes: loosening, stratification, and moving through the sieve, while The looseness of the particles determines the stratification effect, the better stratification effect the higher screening efficiency. Therefore, the degree of the loosening and stratification of the particles directly determines the screening efficiency.

Using the coordinate system shown in Fig 2 as an example, the elliptical vibrating screen is formed in the *xoy* plane with an elliptical vibration [20], which is a vibration in a plane with two sub-vibrations as well, with one along the *y*-axis and the other along the *x*-axis, while the sub-vibrations along the *y*-axis can give the particles vertical directions of impact, but in the *z*-axis direction there are no force effects, so the stratification effects are limited. On the contrary, the composite vibrations, as shown in Fig 5, are spatial and three-dimensional, and their in the *xyz* three-coordinate axis directions can be obtained as sub-vibrations, with their particle force being more complex, and their particle systems are looser and more conducive to the delamination of the particles.

The sub-vibration in the direction of *S*_2_ has a vibration direction angle with the screen surface; according to the theory of material throwing [26], under the excitation of *S*_2_, the material carries out the throwing motion on the screen surface for the transportation of the material. The sub-vibration in the direction of *S*_1_ will give a force to the particles along the *z*-axis, which is mainly in charge of the loosening of the particles and plays a vital role in the sieving results.

## 3 Simulation parameter settings and analysis

### 3.1 Simulation parameter settings

Coal was used as the research object; according to the research of Chen, et al. [27] and Zhao, et al. [21], the physical and contact parameters for the material were both set as shown in Table 1. The simulated particles are non-spherical particles aggregated from three spheres, which were themselves divided into three classes according to their granularity: easy-to-sieve particles, difficult-to-sieve particles, and blocks particles, where the easy-to-sieve particles had a relative granularity (ratio of the particle diameter to the sieve pore diameter) of 0.2–0.7 and accounted for 50%, difficult-to-sieve particles had a relative granularity of 0.7–1 and accounted for 30%, and blocks particles with a relative granularity greater than 1 accounted for 20%. Various particles were randomly generated according to their relative particle size classes, totaling 1000 particles per second. The simple harmonic vibrations were respectively set with the *S*_1_ and *S*_2_ amplitudes and frequencies of 9Hz and 1.5mm, and 18Hz and 1.5mm, the Rayleigh time step was 1.5×10^-6^s, and the simulation time(T) was 20 seconds. At the same time and under the same test conditions, a linear vibrating screen with a frequency of 18Hz, amplitude of 1.5mm, and a vibration direction angle of 45° was set as a contrast test.

**Table 1:** Physical and contact material parameters.

Parameter	Value	
	Sieves	Coal
Density (kg/m^3^)	7580	1600
Poisson’s Ratio	0.25	0.30
Shear Modulus (GPa)	79.92	1.00
Coefficient of Restitution	0.5	0.5
Coefficient of Static Friction	0.4	0.6
Coefficient of Rolling Friction	0.05	0.05
Screen size(mm)	100×250	
Perforating ratio(%)	49.58	
Aperture size(mm)	5×5 (square)	

### 3.2 Analysis of simulation results

**Fig 6:**
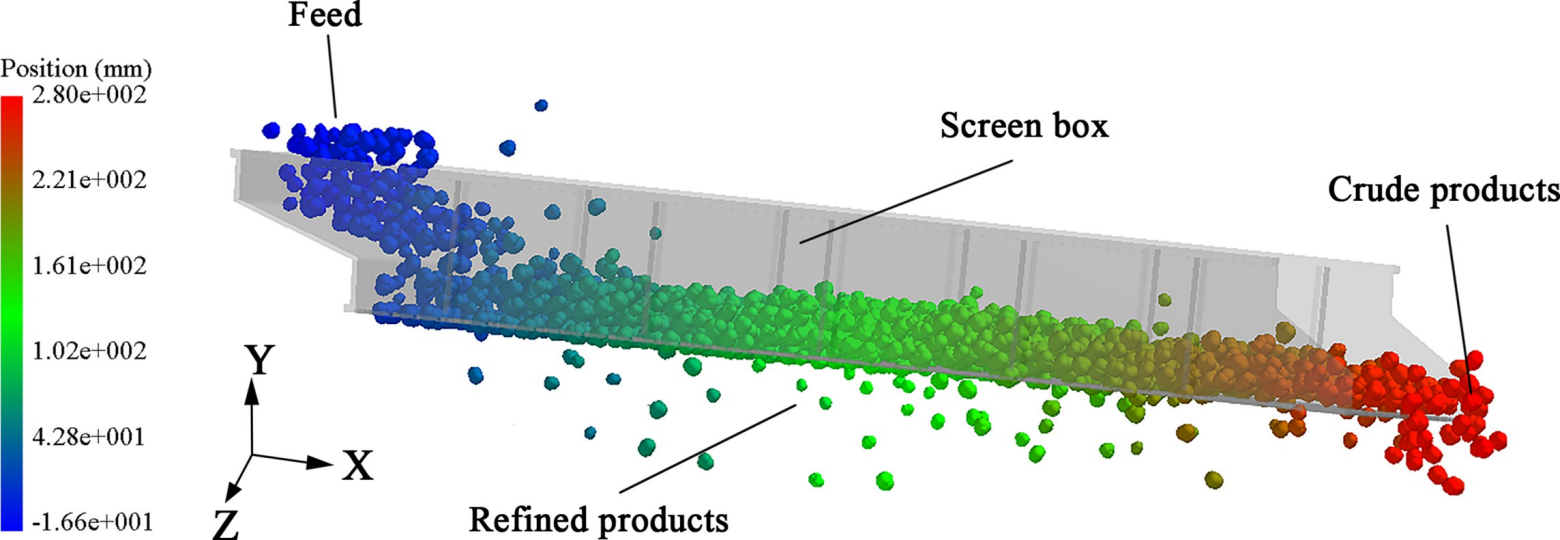
Sieving effect diagram.

**Fig 7:**
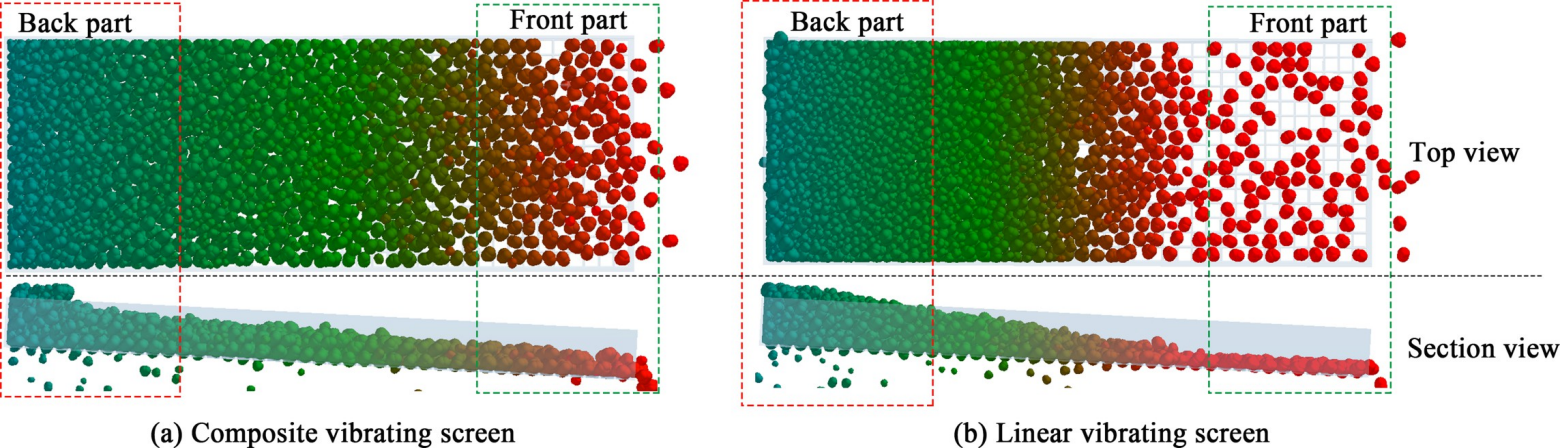
Composite vibrating screen and linear vibrating screen particle motion diagram.

The material sieving process is shown in Fig 6. To understand the movement of the particles on the screen’s surface, the particle distribution for the composite vibrating screens was compared with those of linear vibrating screens, as shown in Fig 7.

From the Fig 7(B) top view, it can be observed that the particles distribution at the front and back parts of the linear vibrating screen is very uneven, and the number of particles at the front part is very small, while the particles at the back part of the screen body have aggregated; from the sectional view, it can be seen that there are huge differences in the thicknesses of the material layers between the front part and the back parts of the screen box, which is not conducive to the transportation of the material. On the contrary, in the composite vibrating screen, Fig 7(A), the particles’ distribution on the screen’s surface is more uniform, and the material layer’s thickness front and back the screen box is also more uniform.

**Fig 8:**
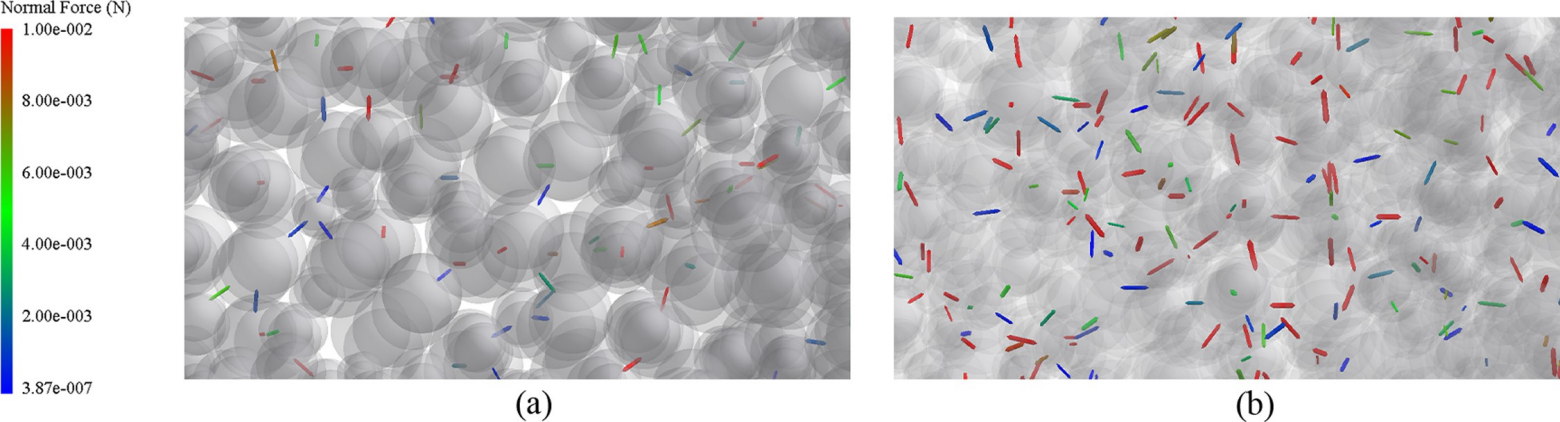
The internal contact forces of the particles.

To explain this phenomenon, the internal contact forces of the particles were extracted; as shown in Fig 8, the bonds with colors represent the interaction forces between the particles, with deeper colors representing greater forces. From Fig 8(A), it can be seen that the force bonds between the composite vibrating screen particles are both fewer and weaker with no network structures formed, and the forces between particles are weaker. From Fig 8(B), it can be seen that the force bonds under the linear vibrating screen are both denser and interconnected, forming dense network structures, strengthening the stability of the particles and making it more difficult for external influences to break their stable internal structures.

Calculate the throwing index *D*_*K*_ of the vibrating screen according to equation 11, where when *D*_*K*_ is more than 1, the material is thrown up with a throwing motion, and when it is less than 1, the material cannot be thrown up [26].


$$D_{K}=\frac{4A\pi^{2}f^{2}\sin\delta }{g\cos\alpha_{0}}$$ (11)

From the calculation, it can be seen that the throwing index of the linear vibrating screen is 1.38, the throwing index of the material can only undergo a slight throwing motion (with 1<*D*_*K*_<1.75), and with the accumulation of the force of gravity after the aggregation of the particles, the internal force chain structures of the particles are relatively solid [28], as seen in the analysis in Fig 8(B); the linear vibrations provide a single impact force, damage to the force chain structure is limited, and the energy provided by the screen is not sufficient to break the strong chain structures inside the particles, so the material cannot undergo a larger throwing action, and can only rely on the friction provided by the screen as well as the force of gravity for its movement, so ultimately, its discharge speed is less than its feed speed, and the material accumulation, while in turn, this seriously affects both the efficiency and transportation of the screening.

**Fig 9:**
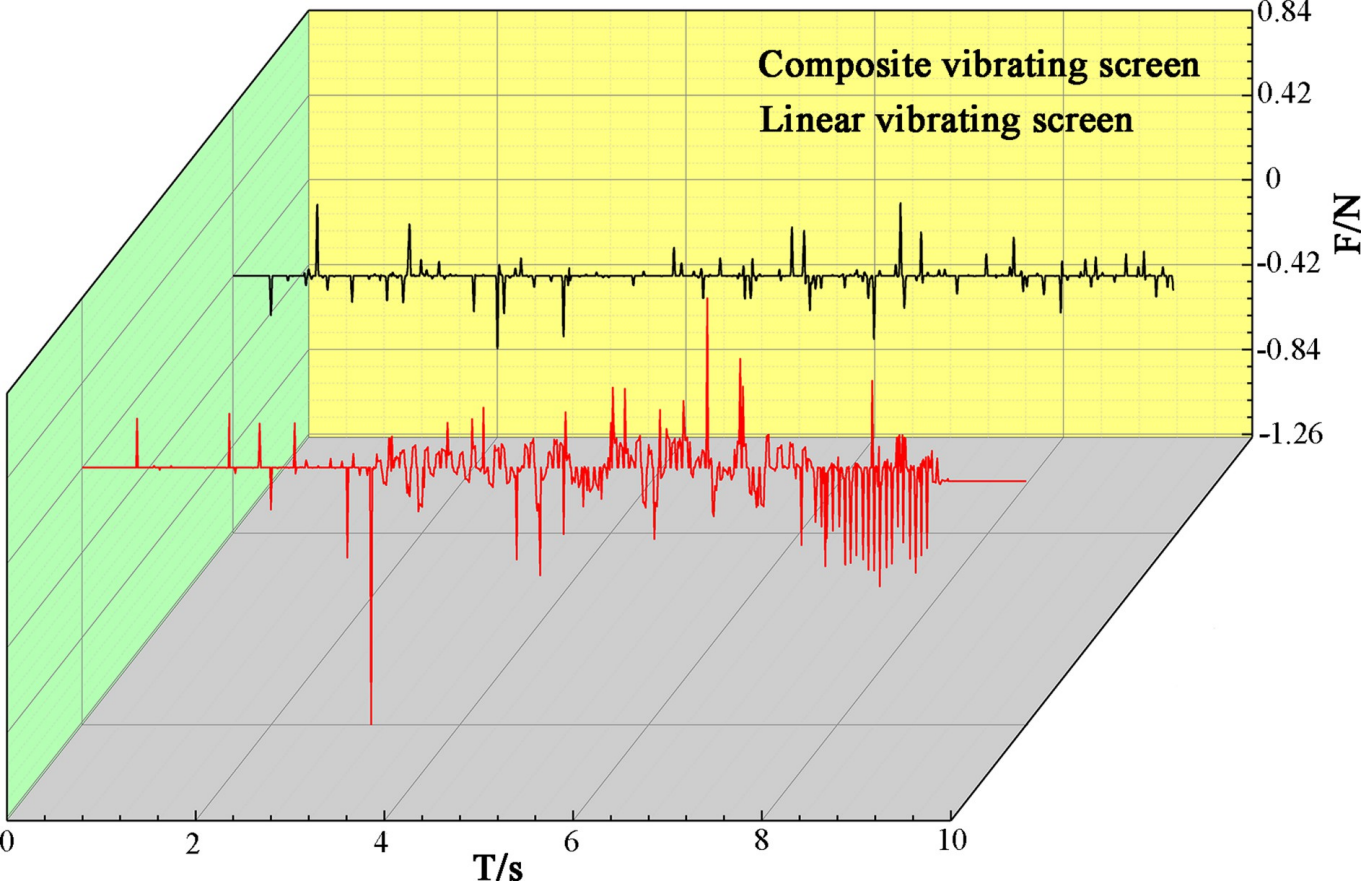
Particle force diagram in the *z*-axis direction.

At the same time, the composite vibrating screen throwing index was also 1.38, and the particles on the composite vibrating screen did not accumulate under the same throwing index. This was due to sub-vibrations in the composite vibrations in the *S*_1_ direction, which play a crucial role in both the loosening and stratification of the material. By extracting the forces of the particles in the direction of the *z*-axis, as shown in Fig 9, it can be seen that the particles in the direction of the *z*-axis under composite vibrations are larger than those under linear vibrations, the particles in this direction of the movement trend is greater, and the forces of the particle population are more diversified. In conjunction with Fig 8(A), it can be seen that composite vibrations can effectively break strong chain structures that are generated by gravitational accumulation, increase the internal looseness of particle systems, and allow the particles to have sufficient space to move within the group. According to "void filling" mechanisms [29]: collisions occur after the particles are excited by vibrations, resulting in the creation of voids, and the particles are then subject to gravity and other factors in their falling process, with smaller particles having a greater chance of falling into such voids, forcing larger particles upward, and then completing the layering of the particles and forming a "Brazil nut" configuration.

**Fig 10:**
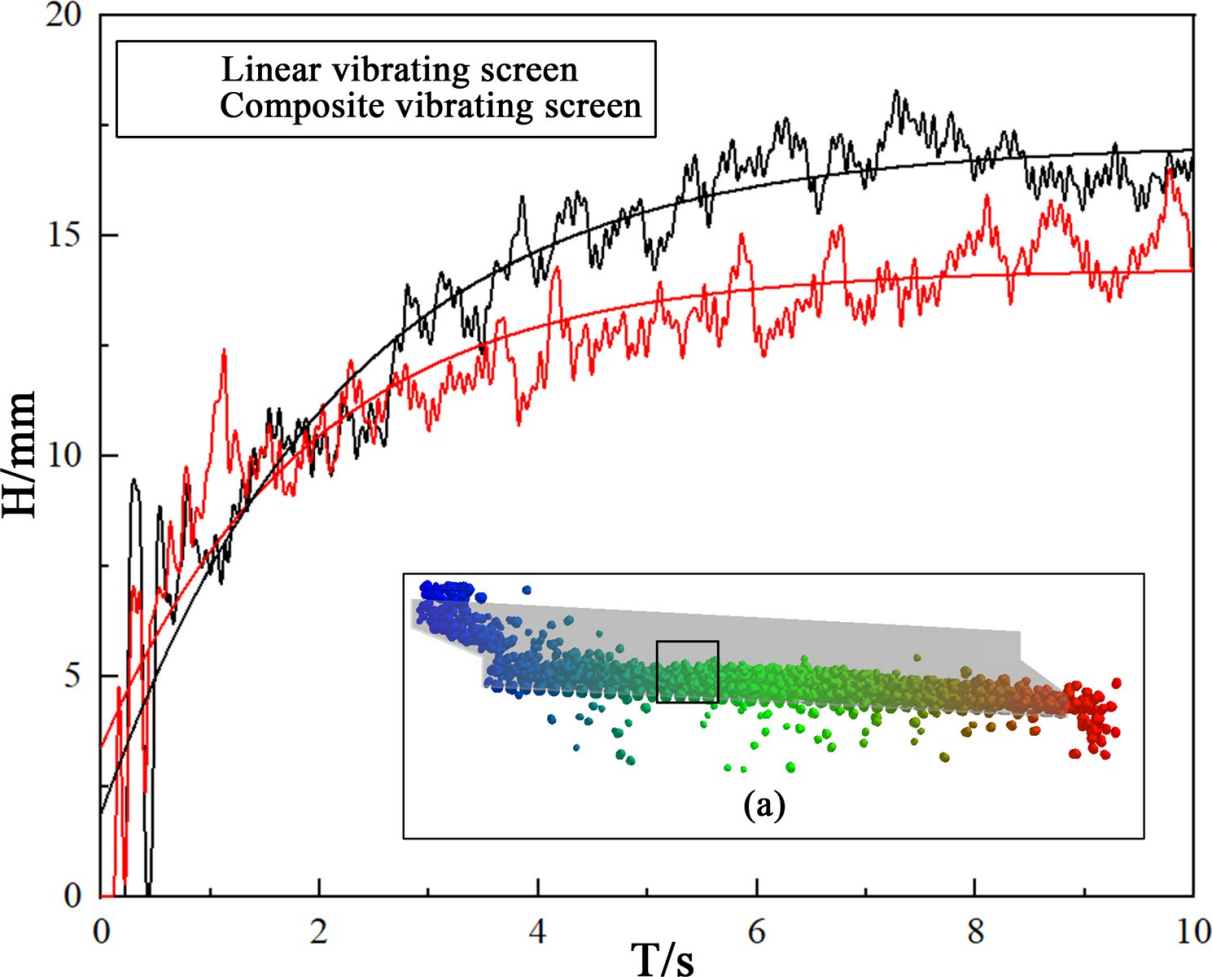
The heights of the screenable particles.

In order to understand the particle stratification for two vibrating screens, as shown in Fig 10(A), the grid selection function in the EDEM software was used for extracting the height changes of the screenable particles(Particle diameter smaller than sieve pore diameter) within the grid.

It can be seen from Fig 10 that the heights of the screenable particles in the linear vibrating screens are greater than those in the composite vibrating screens, which means the composite vibrating screens were more likely to produce "Brazil nut" configurations, and the screenable particles are closer to the screening surface, allowing them to more easily move through sieves.

From analyzing Figs 9 and 10, it can be seen that composite vibrations due to the presence of two sub-vibrations provide different directions of forces to the particle material, unlike the linear vibrating screens where only one direction of force exists. So the force chain generated by gravity becomes easier to break, while the particle becomes looser, enhancing the internal motion of the particle and promoting the stratification of the particles; the particle thus more easily produces stratifications, improving the screening efficiencies of materials. Due to the existence of the screen inclination angle, loose particles are more likely to move toward the outlets under the action of the partial force of gravity, thus promoting the transportation of materials without accumulation. On the other hand, linear vibrating screens need more energy from the screen to make the material pass through the screen and be transported normally, which inevitably increases the energy consumption of vibrating screens.

**Fig 11:**
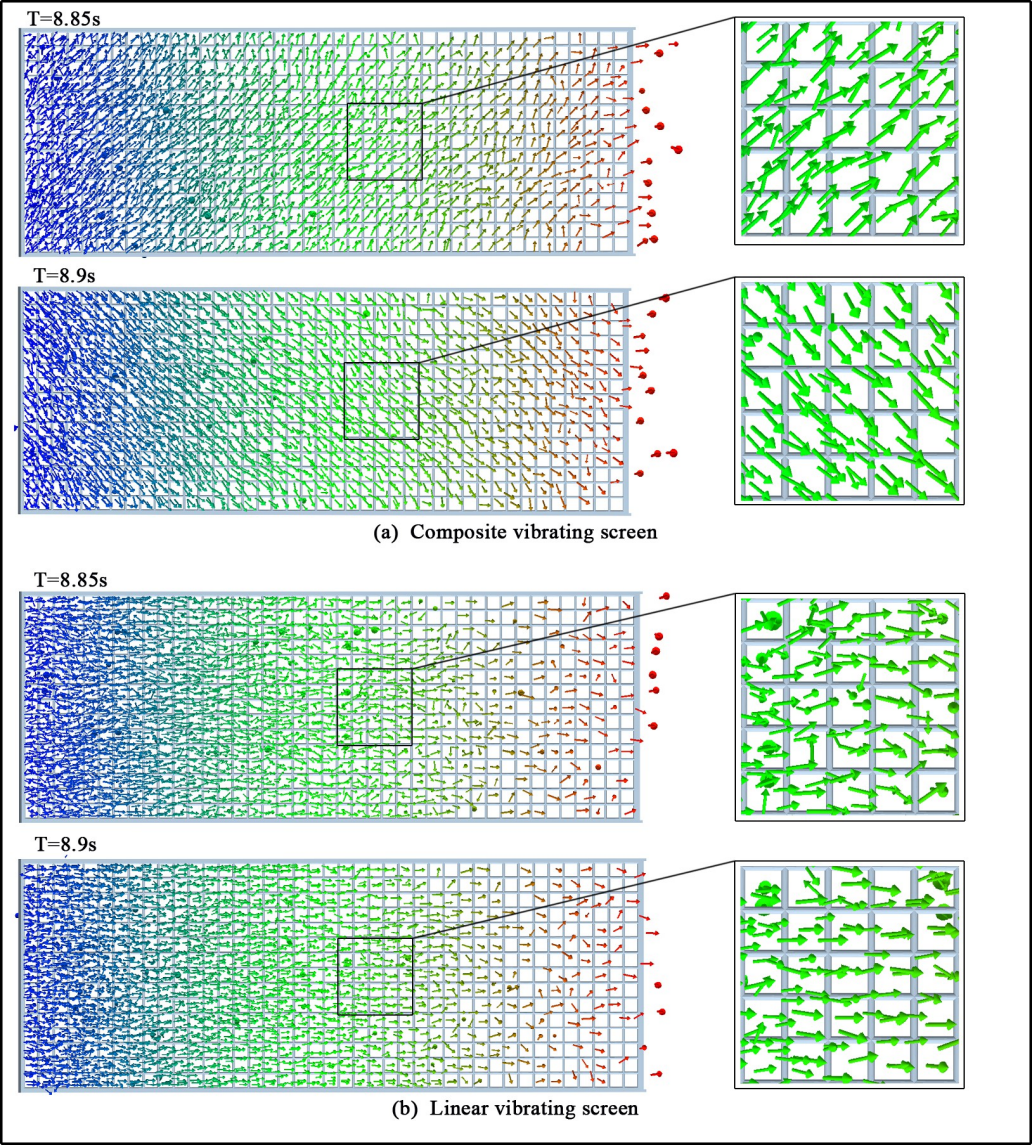
Particle velocity vector.

As seen in Fig 9, the force along the direction of the *z*-axis on the composite vibrating screen the particles are subjected to is much larger than that of the linear vibrating screen, so the trajectories of the particles on the two kinds of vibrating screens should also differ. In order to explore this issue, the velocity vector diagrams of the particles are extracted, as shown in Fig 11. From the picture, we can see that due to the existence of excitation forces along the *z*-axis, the motions of the particles on the composite vibrating screen are to the left front and then to the right front. In contrast, the particles on the linear vibrating screen generally move forward along a straight line. This special motion behavior of the particles on the composite vibrating screen inevitably increases the lengths of the motion paths of the particles on it.

**Fig 12:**
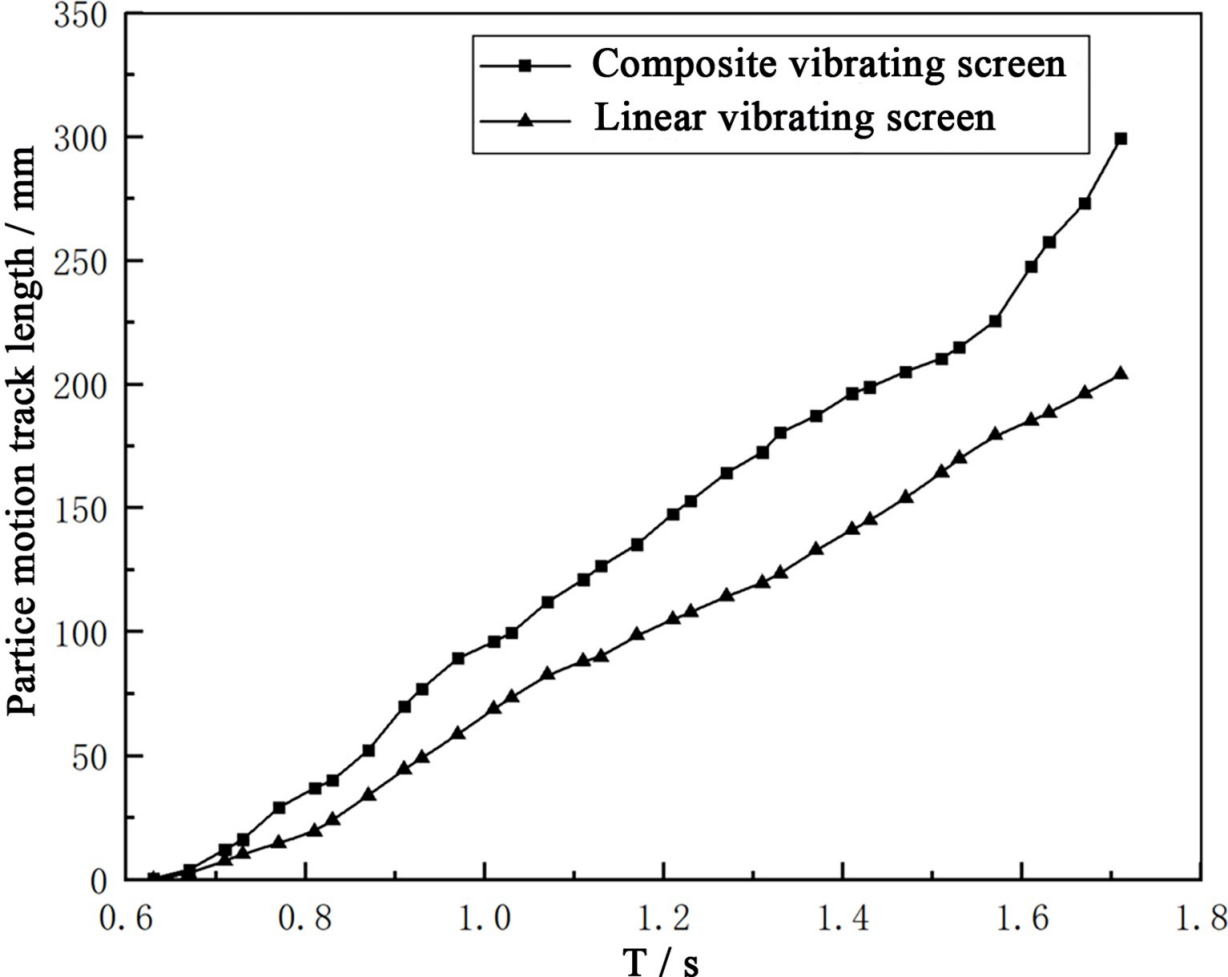
Particle motion length diagram.

In order to obtain a complete count of the path lengths of the particles moving from the feed process to the discharge process, the path lengths for the blocks particles in the two vibrating screens were extracted, as shown in Fig 12. Under the same screen surface lengths, the particle motion path lengths were larger under composite vibrations. Similarly, it can be deduced that the motion paths of the screenable particles on the vibrating screen also become longer than those on linear vibrating screens.

Wang et al. investigated the relationship between the screening efficiency and the screen length, and concluded that the screen length is directly proportional to the screening efficiency [30]. The particles on the screen surface have large movement distances; this is also equivalent to an increase in the length of the screen’s surface, which also promotes improvements in screening efficiency. In other words, at the same screening efficiency, the screen surfaces of composite vibrating screens can be made shorter, greatly reducing the vibration quality of the screen box, thus reducing energy consumption.

**Fig 13:**
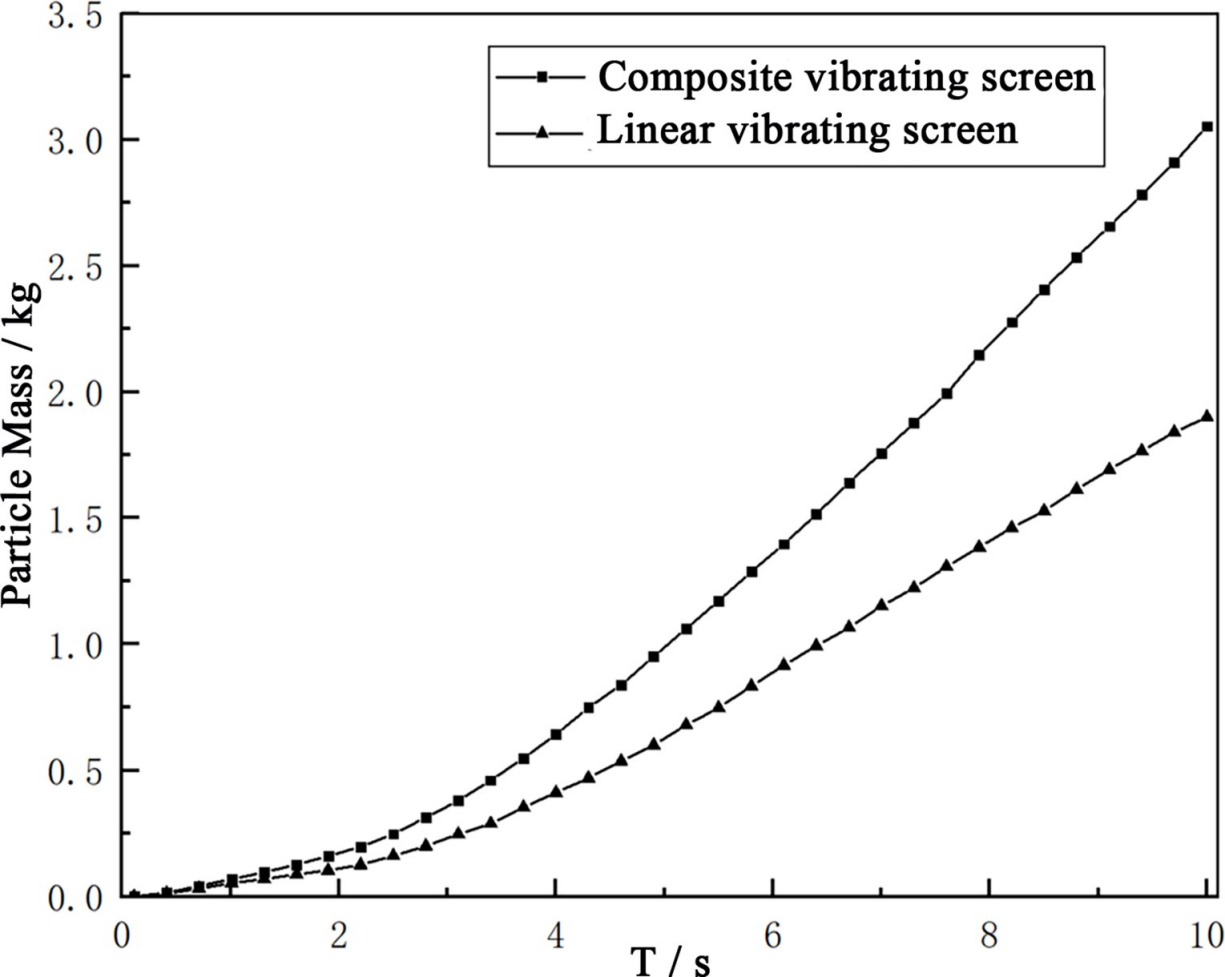
Transportation capacity chart.

The longer paths of the particles moving on the screen surface may result in an increase in the movement times of the particles on the screen surfaces, which may cause the transportation capacities of the vibrating screens to weaken. In order to quantify the advantages and disadvantages of the two in terms of their material transportation capacities, a grid was used to count the masses of the first 10s of both crude and refined products. The greater the mass, the greater the transportation capacity of the vibrating screen. As shown in Fig 13, The material transportation capacity of composite vibrating screen is 1.61 times that of linear vibrating screen, thus it is understood that composite vibrating screens have stronger material transportation capacities under identical vibration parameters.

The particles on the screen surface movement path are long, but the composite vibrating screen transportation capacity is strong because the particles on the composite vibrating screen are more active, their mobility is strong, and it is conducive to the discharge of the screen surface.

The screening efficiency equation is defined as [31, 32]:

$$\eta =\frac{(\alpha -\gamma )(\beta -\alpha )\times 100}{\alpha (\beta -\gamma )(100-\alpha )}\times 100\%$$ (12)

Where: *η* is the screening efficiency as a percentage, *α* is the content of the material smaller than the sieve pore diameter in the feeding material as a percentage, *γ* is the content of the material smaller than the sieve pore diameter in crude products as a percentage, and *β* is the content of the material smaller than the sieve pore diameter in refined products as a percentage.

The screening efficiency is an important indicator used to quantify the screening quality, and for comparing the screening efficiencies of two kinds of vibrating screens while directly observing the performance of both. From the Fig 7, it can be seen that linear vibrating screens under the vibration parameter of *f* = 18Hz, *A* = 1.5mm, the material is piled up on the screen surface, which can’t complete the screening operation. Therefore, it is necessary to appropriately increase the amplitude of the linear vibrating screen, using 2mm, 2.5mm, 3mm for the three simulations. It has been found that the linear vibrating screen amplitudes of 2mm and 2.5mm will occur when the accumulation of material cannot complete the screening; when the amplitude is increased to 3mm, the screening work can be completed. Therefore, the screening efficiencies of linear vibrating screens with the vibration parameters 18Hz and 3mm and those of composite vibrating screens of 18Hz and 1.5mm were compared. In order to ensure the accuracy of the calculations, the instantaneous sieving efficiencies for 5s, 10s, and 15s of simulations, as well as the total screening efficiency of the whole simulation process were selected for comparison.

**Fig 14:**
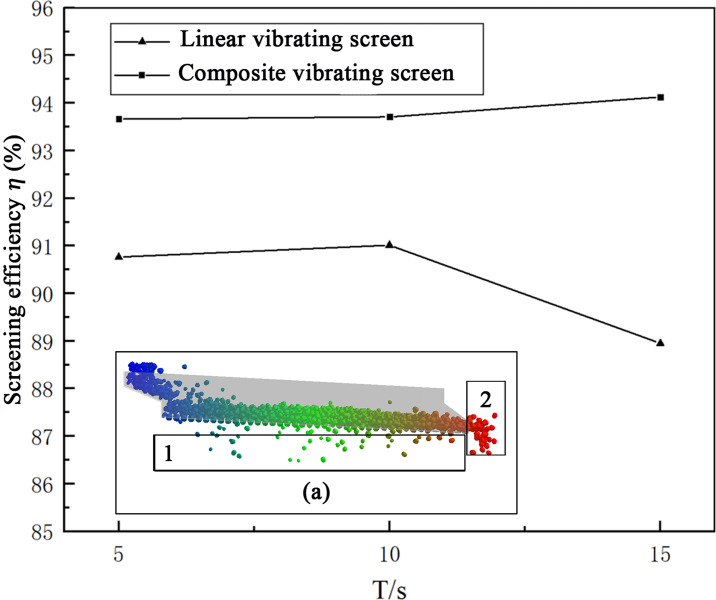
Screening efficiency graph.

Using the grid 1 and grid 2 statistics for the masses of particles of the crude and refined products, as shown in Fig 14(A), it can be seen from the picture that the instantaneous screening efficiencies of composite vibrating screens were 3–4% higher than those of linear vibrating screens. During the whole simulation process, the composite vibrating screen screening efficiency was calculated as 93.75%, the linear vibrating screens’ screening efficiency was calculated as 90.7%, and the composite vibrating screen was calculated as 3.05% higher. It can be seen that composite vibrating screens can achieve better screening effects than linear vibrating screens with smaller vibration parameters, indicating the advantages of composite vibrating screens.

## 4 Validation experiments

In order to verify whether the screening effects are improved after adding a *z*-axis excitation force, the experimental platform shown in Fig 3B was used for coal screening experiments. When only two high-frequency excitation motors are working, the platform is equivalent to a linear vibrating screen, as there is no z-axis excitation force; four excitation motors working at the same time can add a *z*-axis excitation force to the screen body. The governor used to control the frequency ratio of the high frequency excitation motor and low frequency excitation motor was 2:1.

Using 28g of large particles and 115g of small particles, the particle mixture was created. The mixed particles were poured uniformly from the feed part of the sieve body and the crude and refined products were collected separately, and the masses of the coarse and fine products were obtained using an electronic scale.

**Fig 15:**
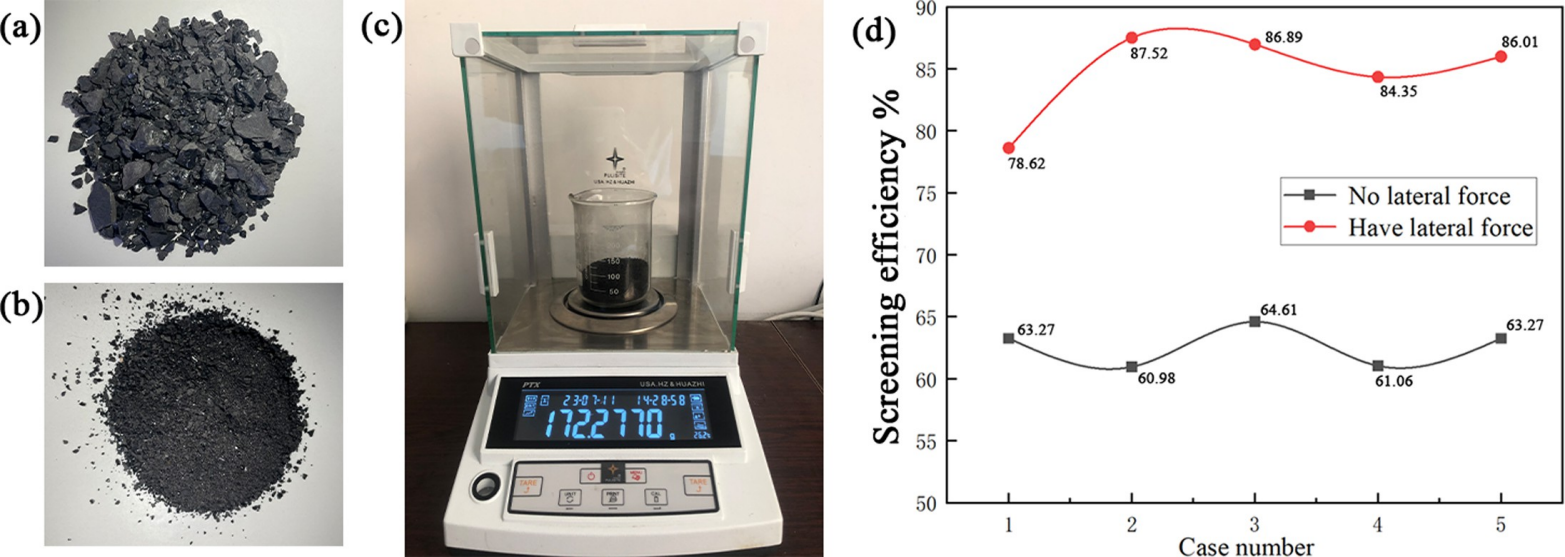
*z*-axis excitation force validation experiment (a is coal particles with particle sizes larger than the sieve pore diameter, i.e., large particles; b is coal particles with particle sizes smaller than the sieve pore diameter, i.e., small particles; c is the electronic scale; d is the data obtained from the experiment).

Conducted 5 sieving experiments, the screening efficiency obtained from the experiment is shown in Fig 15; the average value of the screening efficiency without a z-axis excitation force was 62.638%, and the screening efficiency after adding *z*-axis excitation force was 84.678%, which was a 22.04% increase in the average screening efficiency. Although the validation experiment could not precisely control the particle shapes, particle sizes to achieve the same effects as the numerical simulation, it proved that there was a large improvement in the screening efficiency after adding a *z*-axis excitation force, indicating that it is feasible to improve the screening efficiency by adding a *z*-axis excitation force.

## 5 Conclusions

In this study, two mutually perpendicular one-dimensional simple harmonic vibrations with a 1:2 frequency ratio were synthesized as Lissajous composite vibrations, and the motion behaviors of the material particles on the screen surfaces were studied, leading to the following conclusions:

1. The simulation study found that using a composite vibrating screen for material screening was feasible, and offered greater advantages in terms of screening efficiency and material transportation. Under the same vibration parameters, the transportation capacity of the composite vibrating screen material was stronger, and its same-time processing volume was 1.61 times that of the linear vibrations; the screening efficiency of composite vibrating screens was 93.75%, while that of the linear vibrating screens was 90.7%, an increase of 3.05%.
2. The sub-vibrations in the *S*_2_ direction were responsible for the transportation of materials, while the sub-vibrations in the *S*_1_ direction played a vital role in the screening process; due to the existence of vibrations in this direction, the forces in the material particles in this direction increased. The screen surfaces can provide *xyz* three directions of vibration forces to the particles, which effectively breaks the internal force chain structures of the particles so that the looseness of the material particles increased, which was conducive to forming "Brazil fruit" configurations and strengthening the screening effects.
3. Compared with linear vibrating screens, the distributions of particles on the screen surfaces under composite vibrations are more uniform, which improves the screen surface utilization area; at the same time, the movement paths of the particles on the screen surface were longer, which increases their probability of penetrating the screens.
